# Phosphorylation of Tip60 by p38α regulates p53-mediated PUMA induction and apoptosis in response to DNA damage

**DOI:** 10.18632/oncotarget.2717

**Published:** 2014-12-10

**Authors:** Yingxi Xu, Rong Liao, Na Li, Rong Xiang, Peiqing Sun

**Affiliations:** ^1^ College of Medicine, Nankai University, Tianjin, P.R. China, 300071; ^2^ Departments of Cell and Molecular Biology, The Scripps Research Institute, La Jolla, CA 92037

**Keywords:** p38, Tip60, p53, PUMA, apoptosis

## Abstract

Tip60 is a multifunctional acetyltransferase involved in multiple cellular functions. Acetylation of p53 at K120 by Tip60 promotes p53-mediated apoptosis after DNA damage. We previous showed that Tip60 activity is induced by phosphorylation at T158 by p38. In this study, we investigated the role of p38-mediated Tip60 phosphorylation in p53-mediated, DNA damage-induced apoptosis. We found that DNA damage induces p38 activation, Tip60-T158 phosphorylation, and p53-K120 acetylation with similar kinetics. p38α is essential for DNA damage-induced Tip60-T158 phosphorylation. In addition, both p38α and Tip60 are essential for p53-K120 acetylation, binding of p53 to PUMA promoter, PUMA expression and apoptosis induced by DNA damage. Moreover, DNA damage induces protein kinase activity of p38α towards Tip60-T158, and constitutive activation of p38 in cells leads to increases in Tip60-T158 phosphorylation, p53-K120 acetylation, PUMA expression and apoptosis. Furthermore, the Tip60-T158A mutant that cannot be phosphorylated by p38 fails to mediate p53-K120 acetylation, PUMA induction, and apoptosis following DNA damage. These results establish that Tip60-T158 phosphorylation by p38 plays an essential role in stimulating Tip60 activity required for inducing the p53-PUMA pathway that ultimately leads to apoptosis in response to DNA damage, which provides a mechanistic basis for the tumor-suppressing function of p38 and Tip60.

## INTRODUCTION

Both chemotherapy and radiotherapy can induce DNA damage in rapidly dividing cancer cells, which triggers cell cycle arrest and apoptosis by promoting activation of the p53 signaling pathway. Activated p53 mediates reversible cell cycle arrest through transcriptional induction of p21^WAF1^, thus allowing time for the repair of damaged DNA and subsequent resumption of cell proliferation upon the completion of DNA repair [1–2]. On the other hand, cells that are unable to repair damaged DNA undergo apoptosis, a process that is also mediated by p53. Upon posttranslational modification, p53 mediates apoptosis by inducing the transcription of PUMA [3–4]. PUMA is a pro-apoptosis member of the BH3-only subgroup of the Bcl-2 family [5], whose proapoptotic activity requires the interactions with other Bcl-2 family members and mitochondria localization [6]. PUMA could bind to Bcl-2, induce the activation of multi domain proapoptotic protein Bax and/or Bak, localize to the mitochondria to induce cytochrome c release, and trigger mitochondria dysfunction and caspase activation, thereby activating the rapid induction of programmed cell death [7].

The mechanism by which p53 transactivates different sets of target genes to result in either cell-cycle arrest or apoptosis is not well understood. The transcriptional activity of p53 is regulated by various posttranslational modifications [8], which might impact the decisions of cell fate. In addition to phosphorylation, p53 is acetylated in response to DNA damage, and the level of acetylation contributes to p53 activation [9–11]. p53 can be acetylated by the histone acetyltransferase CBP/p300 and p300/CBP associated factor (PCAF) at K320 [10, 12], K164 [13] or C-terminal domain (K370, 372, 373 and K382) [11], which blocks Mdm2 and Mdmx binding to p53, thus preventing degradation of p53 and promoting the recruitment of p53 to target promoters. In addition, p53 can be acetylated at K120 by acetyltransferase hMOF and Tip60, which can induce the proapoptotic activity of p53, but has no effect on the ability of p53 to mediate cell cycle arrest [14].

Tip60 is a member of the MYST family of histone acetyltransferases (HATs), which has been indicated in several cellular processes [15–16]. Tip60 can act as a transcriptional coactivator after being recruited to target promoters and enhance transactivation of target genes through acetylation of histones. Furthermore, Tip60 can modify the activity or expression of non-histone substrates through direct interaction and acetylation in a transcription-independent mechanism [14–16]. Tip60 has been shown to participate in apoptosis [17–18], DNA damage responses [19], and oncogene-induced senescence [20], and is a potential tumor suppressor [21]. Despite the critical function of Tip60 in several cellular processes, the upstream signaling pathways that regulate theTip60 acetyltransferase activity have been poorly studied. We previously showed that in response to activation of the *ras* oncogene, p38α phosphorylates Tip60 at T158 to induce its acetyltransferase activity and function in oncogenic *ras*-induced senescence [20]. This finding prompted us to investigate whether p38-mediated phosphorylation of Tip60-T158, which activates the acetyltransferase activity of Tip60, also contributes to other biological functions of Tip60, such as DNA damage-induced apoptosis. In the current study, we found that DNA damage induces Tip60-T158 phosphorylation in a p38α-dependent manner. Both p38α and Tip60 are required for DNA damage-induced p53 acetylation at K120, and subsequent binding to p53 to the PUMA promoter and transactivation of PUMA gene expression, and are essential for DNA damage-induced apoptosis. Moreover, DNA damage induces the protein kinase activity of p38 towards Tip60, and constitutive activation of p38 in cells leads to increases in Tip60-T158 phosphorylation, p53-K120 acetylation, PUMA expression and apoptosis. Furthermore, whereas wild type murine Tip60 restored DNA damage-induced p53-K120 acetylation, PUMA expression and apoptosis in cells expressing human specific Tip60 shRNA, the mouse Tip60 mutant that cannot be phosphorylated by p38 (T158A) failed to do so. These results demonstrate that p38-mediated Tip60-T158 phosphorylation contributes to the ability of Tip60 to mediate p53 acetylation and activation, PUMA expression and apoptosis induction in response to DNA damage. These findings have established an essential role of the p38-Tip60-p53-PUMA pathway in DNA damage triggered apoptosis.

## RESULTS

### Tip60 is phosphorylated at T158 with same kinetics as p38 activation in response to DNA damage

**Figure 1:**
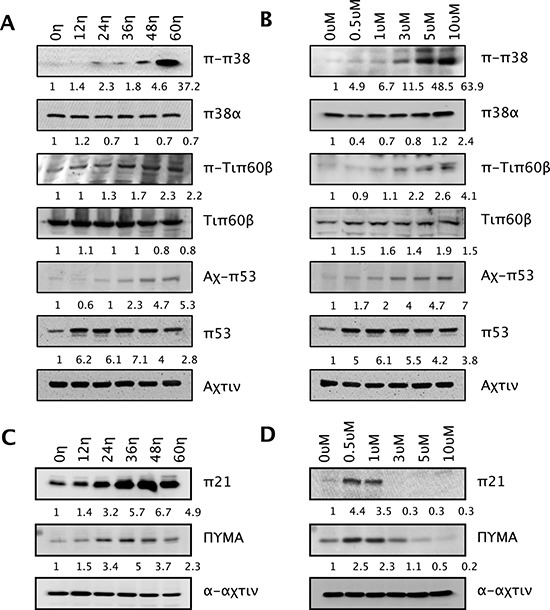
p38 activation, Tip60-T158 phosphorylation, and p53-K120 acetylation are induced with same kinetics by DNA damage **(A)** Weston blot analysis of U2OS cells treated with 1 μM of Dox for indicated durations, detecting p38α, p–p38, p-Tip60-T158, Tip60, ac-p53-K120, p53 and actin. **(B)** Weston blot analysis of U2OS cells treated with indicated concentrations of Dox for 24 h, detecting p38α, p-p38, p-Tip60-T158, Tip60, ac-p53-K120, p53 and actin. **(C)** Weston blot analysis of U2OS cells treated with 1 μM Dox for indicated durations, detecting PUMA, p21 and actin. **(D)** Weston blot analysis of U2OS cells treated with indicated concentrations of Dox for 24 h, detecting PUMA, p21 and actin.

To investigate the involvement of p38-mediated Tip60 phosphorylation during DNA damage, U2OS cells were first treated with Doxorubicin (Dox), a chemotherapeutic drug that induces DNA double-strand breaks [22]. Dox treatment induced both activating phosphorylation of p38 and phosphorylation of Tip60 at T158 with similar kinetics in a time-dependent (over a duration from 12 to 60 hours at 1 μM) and dose-dependent (with concentrations ranging from 0.5 to 10 μM) manner (Figure 1A and 1B).

Given the ability of Tip60 to acetylate p53 at K120 [14, 23–24], we analyzed changes in the expression of p53 acetylated at K120 (Ace-K120-p53) after Dox treatment. As expected, expression levels of Ace-K120-p53 increased after treatment with Dox, with the same kinetics as the induction of p38 phosphorylation and Tip60 phosphorylation (Figure 1A and 1B).

Furthermore, consistent with the induction of p21^WAF1^ [2] and PUMA [18] by activated p53 following DNA damage, the expression of p21^WAF1^ and PUMA were increased in a time-dependent manner after Dox treatment (Figure 1C). However, in the dose-depended test, the induction of p21^WAF1^ and PUMA was maximal at 1 μM of Dox, followed by a sharp decrease at concentrations of 3 μM and above (Figure 1D). The reduction in p21^WAF1^ and PUMA expression by high concentrations of Dox was reported before [1, 25–26]. However, the mechanism behind this observation is unclear. One possible explanation is that high Dox concentrations induce strong apoptosis leading to selection against high p21^WAF1^- and PUMA-expressers in the cell populations subjected to analysis. Alternatively, high Dox concentrations may induce p53-independent mechanisms that inhibit the expression of p21^WAF1^ and PUMA. We had thus performed further analyses using 1 μM of Dox.

### p38α is required for phosphorylation of Tip60 at T158, Tip60-mediated acetylation of p53 at K120, and p53-mediated induction of p21^WAF1^ and PUMA following DNA damage

**Figure 2:**
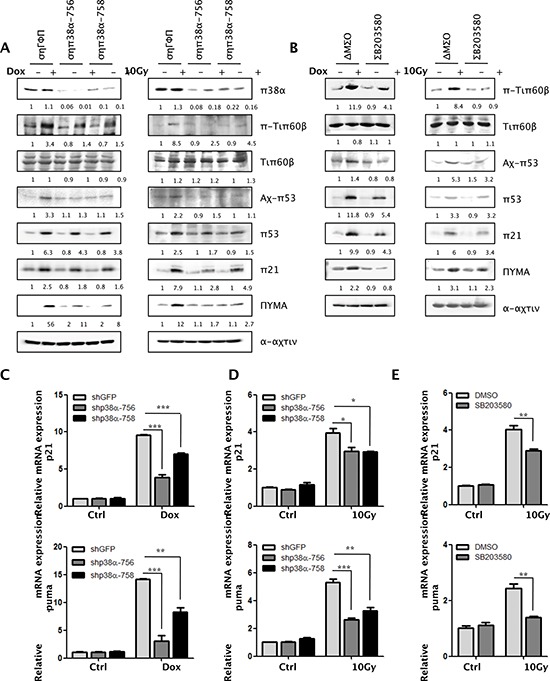
p38α is required for the induction of Tip60-T158 phosphorylation, p53-K120 acetylation and p21^WAF1^ and PUMA expression following DNA damage **(A)** U2OS cells transduced with shRNA for GFP (shGFP) or p38α (shp38α-756 or -758) were treated with 1 μM of Dox for 36 h (left panels) or 10 Gy of γ-radiation followed by incubation for 72 h (right panels). Cell lysis was subjected to Western blot analysis detecting the indicated proteins. **(B)** U2OS cells were pre-treated with 10 μM of the p38 inhibitor SB203580 or DMSO for 1 h, and then treated in the presence of SB203580 or DMSO with 1 μM of Dox for 36 h (left panels) or 10 Gy of γ-radiation followed by incubation for 72 h (right panels). Cell lysis was subjected to Western blot analysis detecting the indicated proteins. **(C–D)** U2OS cells transduced with shRNA for GFP (shGFP) or p38α (shp38α-756 or -758) were treated with 1 μM of Dox for 36 h (C) or 10 Gy of γ-radiation followed by incubation for 72 h (D) mRNA levels of PUMA (lower panels) and p21^WAF1^ (upper panels) were detected by real-time PCR. Values are mean ± SEM for triplicates. **(E)** U2OS cells were pre-treated with 10 μM of the p38 inhibitor SB203580 or DMSO for 1 h, and then treated in the presence of SB203580 or DMSO with 10 Gy of γ-radiation followed by incubation for 72 h. mRNA levels of PUMA (lower panel) and p21^WAF1^ (upper panel) were detected by real-time PCR. Values are mean ± SEM for triplicates.

The finding that p38 activation, Tip60-T158 phosphorylation, and p53-K120 acetylation are induced with similar kinetics after DNA damage (Figure 1) suggests that p38 may phosphorylate Tip60 at T158, leading to activation of Tip60 that in turn acetylates p53 at K120. To determine whether p38 is responsible for Tip60 phosphorylation and activation, we examined the effect of p38 knockdown in U2OS cells during Dox-or γ radiation-induced DNA damage. In contrast to the control, GFP shRNA, the p38α shRNAs (shp38α-756 and shp38α-758) [27] knocked down p38α expression, and greatly reduced the induction of Tip60-K158 phosphorylation and p53-K120 acetylation by Dox and γ-radiation (Figure 2A). Consistent with the ability of p53 to induce PUMA transcription upon acetylation at K120 by Tip60 [14], p38αshRNA also decreased the induction of PUMA protein (Figure 2A) and RNA (Figure 2C and 2D) levels by DNA damage. Furthermore, we examined the effect of a chemical inhibitor of p38, SB203580, which inhibits p38 catalytic activity by binding to the ATP-binding pocket, but does not inhibit phosphorylation of p38 by upstream kinases [28]. We again observed a similar decrease in the induction of Tip60-T158 phosphorylation, p53-K120 acetylation, and PUMA expression by Dox or γ-radiation in cells treated with SB203580, as compared to the vehicle-treated cells (Fig. 2B and 2E). Taken together, these results indicate that in response to DNA damage, p38α is responsible for the phosphorylation of Tip60-T158, which stimulates the activity of Tip60 leading to acetylation of p53-K120 and induction of PUMA.

It was reported that acetylation of p53-K120 by Tip60 only stimulates the ability of p53 to bind to the PUMA promoter and to induce PUMA expression, but has no effect on the ability of p53 to regulate the p21^WAF1^ promoter [14]. However, we observed that the p21^WAF1^ induction by DNA damage was also reduced modestly at both protein and mRNA levels in cells expressing p38α shRNA or treated with SB203580 (Figure 2A, 2C, 2D and 2E), although the effect of p38 inhibition was stronger on PUMA than on p21^WAF1^. This indicates that p38 may also activate an additional, Tip60-independent pathway that stimulates the ability of p53 of induce p21^WAF1^ during DNA damage.

### Tip60 is required for acetylation of p53 at K120 and induction of PUMA expression, but not induction of p21^WAF1^, in response to DNA damage

**Figure 3:**
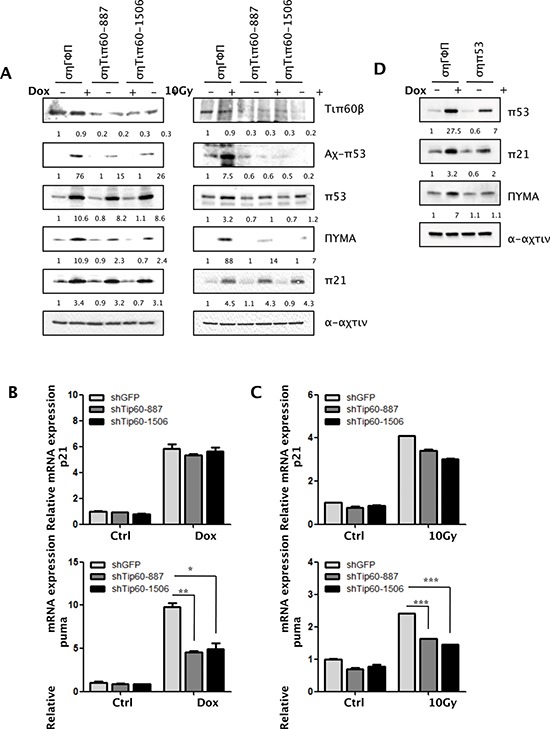
Tip60 is required for acetylation of p53 at K120 and induction of PUMA expression, but not for induction of p21^WAF1^, in response to DNA damage **(A)** U2OS cells transduced with shRNA for GFP (shGFP) or Tip60 (shTip60-887 or -1506) were treated with 1 μM of Dox for 36 h (left panels) or 10 Gy of γ-radiation followed by incubation for 24 h (right panels). Cell lysis was subjected to Western blot analysis detecting the indicated proteins. **(B–C)** U2OS cells transduced with shRNA for GFP (shGFP) or Tip60 (shTip60-887 or -1506) were treated with 1 μM of Dox for 36 h (B) or 10 Gy of γ-radiation followed by incubation for 24 h (C). mRNA levels of PUMA (lower panels) and p21^WAF1^ (upper panels) were detected by real-time PCR. Values are mean ± SEM for triplicates. **(D)** U2OS cells transduced with shRNA for GFP (shGFP) or p53 (shp53) were left untreated (–) or treated with 1 μM of Dox for 24 h (+), and analyzed by Western blotting detecting the indicated proteins.

To further investigate the role of Tip60 in p53 acetylation and activation, we knocked down Tip60 in U2OS cells using shRNAs we previously published [20]. Consistent with previous reports [14, 29], downregulation of Tip60 by shRNAs (shTip60-887 or shTip60-1506) decreased the acetylation of p53 at K120 induced by either Dox or γ-radiation (Figure 3A). Moreover, induction of PUMA expression by DNA damage was decreased at both protein and mRNA levels (Figure 3A, 3B and 3C). Therefore, those findings confirm that Tip60 mediates the acetylation of p53 at K120, which stimulate the p53 activity in inducing a proapoptotic target gene PUMA.

In contrast to the cells expressing p38α shRNAs, neither the mRNA level nor the protein level of p21^WAF1^ changed in cells expressing the Tip60 shRNA (Figure 3A, 3B and 3C). This confirms that Tip60-mediated acetylation of p53 at K120 only influences the transcription of a p53 target gene PUMA that is involved in apoptosis, but not p21^WAF1^ that mediates cell-cycle arrest during DNA damage, whereas p38α regulates the expression of both p53 target genes.

Furthermore, we have confirmed that DNA damage-induced PUMA and p21^WAF1^ expression is p53-dependent, as shRNA-mediated knockdown of p53 abrogated the induction of these 2 genes (Figure 3D). This finding is consistent with previously published results [4].

### p38α and Tip60 are required for apoptosis induction in response to DNA damage

**Figure 4:**
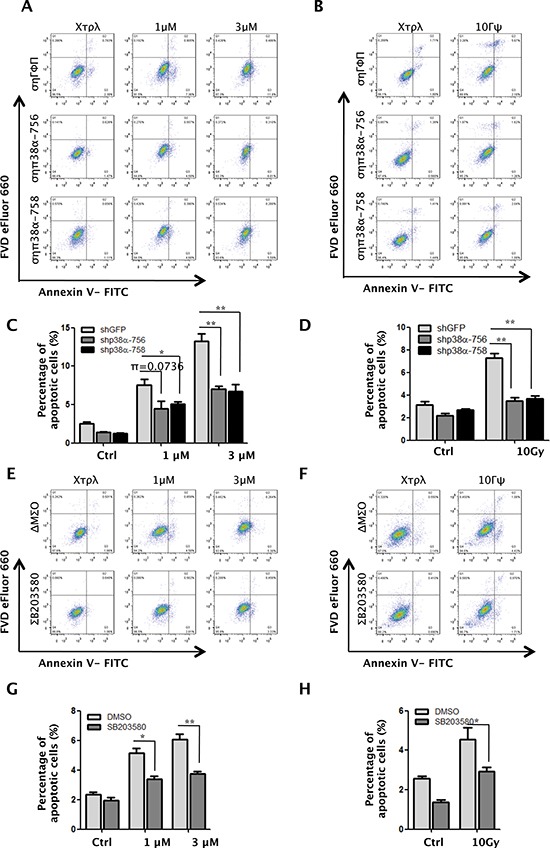
p38α is required for apoptosis induction in response to DNA damage **(A–B)** U2OS cells transduced with shRNA for GFP (shGFP) or p38α (shp38α-756 or -758) were treated with 1 μM or 3 μM of Dox for 24 h (A) or 10 Gy of γ-radiation followed by incubation for 24 h (B). Cells were collected, stained with an FITC-conjugated anti-Annexin-V antibody and FVD eFlour 660 and analyzed by FACS. **(C–D)** Quantification and statistical analysis of the data in A (C) and B (D). The percentage of apoptotic cells was quantified as the percentage of FITC-positive cells in the gated area. Values are mean ± SEM for triplicates. **(E–F)** U2OS cells were pre-treated with the 10 μM of p38 inhibitor SB203580 or DMSO for 1 h, and then treated in the presence of SB203580 or DMSO with 1 μM or 3 μM of Dox for 24 h (E) or 10 Gy of γ-radiation followed by incubation for 24 h (F). Cells were collected, stained with an FITC-conjugated anti-Annexin-V antibody and FVD eFlour 660 and analyzed by FACS. **(G–H)** Quantification and statistical analysis of the data in E (G) or F (H). The percentage of apoptotic cells was quantified as the percentage of FITC-positive cells in the gated area. Values are mean ± SEM for triplicates.

The requirement of p38α in DNA damage-induced Tip60-T158 phosphorylation, p53-K120 acetylation, and PUMA expression raises a possibility that p38α plays an important role in apoptosis induction after DNA damage. We thus analyzed the percentage of Annexin V-positive apoptotic cells by flow cytometry in Dox- and γ-radiation-treated cells with p38α knockdown. When incubated with 1 μM or 3 μM of Dox, approximately 9% or 12%, respectively, of control cells were apoptotic; p38α knockdown reduced the percentage of apoptotic cells to 5% or 7%, respectively (Figure 4A and 4C). Similarly, after 10 Gy of γ-radiation, nearly 8% of control cells were apoptotic; in cells with p38α knockdown, the percentage of apoptotic cells dropped to 4% (Figure 4B and 4D). The p38 inhibitor SB203580 had a similar effect on DNA damage-induced apoptosis. We found that the apoptotic population was decreased in SB203580-treated cells, as compared to the vehicle-treated cells, upon both Dox- and γ-radiation-induced DNA damage (Figure 4E–4H).

**Figure 5:**
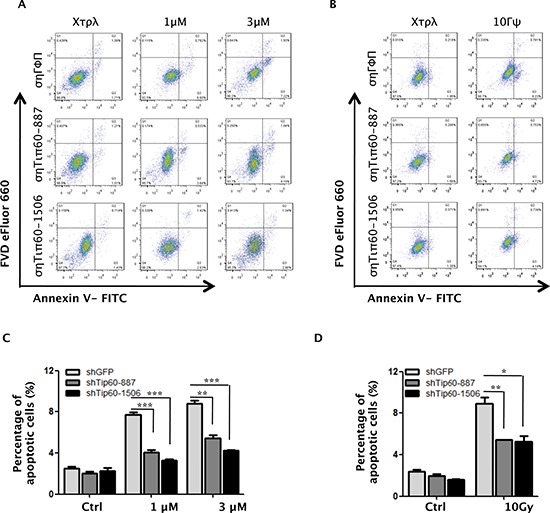
Tip60 is required for apoptosis induction in response to DNA damage **(A–B)** U2OS cells transduced with shRNA for GFP (shGFP) or Tip60 (shTip60-887 or -1506) were treated with 1 μM or 3 μM of Dox for 24 h (A) or 10 Gy of γ-radiation followed by incubation for 24 h (B). Cells were collected, stained with an FITC-conjugated anti-Annexin-V antibody and FVD eFlour 660 and analyzed by FACS. **(C–D)** Quantification and statistical analysis of the data in A (C) or B (D). The percentage of apoptotic cells was quantified as the percentage of FITC-positive cells in the gated area. Values are mean ± SEM for triplicates.

To further investigate the role of the p38-Tip60 pathway in apoptosis induction after DNA damage, we examined the effect of Tip60 knockdown. In cells incubated with 1 μM or 3 μM of Dox for 24 h, we found that Tip60 knockdown reduced the percentage of apoptotic cells from nearly 8% to 4% or from 10% to 6%, respectively (Figure 5A and 5C). Similarly, the percentage of apoptotic cells induced by 10 Gy of γ-radiation was also reduced by Tip60 shRNA (Figure 5B and 5D).

Taken together, these results indicate that both p38α and Tip60 are essential for DNA damage-induced apoptosis, thus implicating a critical function of p38-mediated Tip60 phosphorylation in this biological process.

### p38α and Tip60 are essential for DNA damage-induced binding of p53 to the PUMA promoter

**Figure 6:**
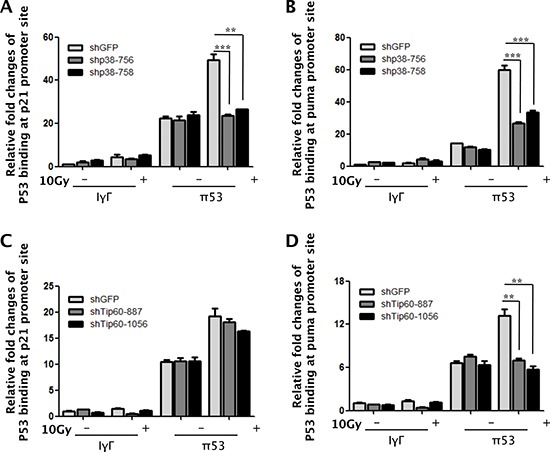
p38α and Tip60 are essential for DNA damage-induced binding of p53 to the PUMA promoter **(A–B)** U2OS cells transduced with shRNA for GFP (shGFP) or p38α (shp38α-756 or -758) were treated with 10 Gy of γ-radiation (10Gy) or left untreated (Ctrl) followed by incubation for 72 h. Cells were lysed and subjected to ChIP using normal mouse IgG (IgG) or a mouse anti-p53 antibody (p53). Immunoprecipitated DNA was used as the template for real-time PCR quantification of the p53-binding sites on the p21^WAF1^ (A) or PUMA (B) promoters. Values are mean ± SEM for triplicates. **(C–D)** U2OS cells transduced with shRNA for GFP (shGFP) or Tip60 (shTip60-887 or -1506) were treated 10 Gy of γ-radiation (10Gy) or left untreated (Ctrl) followed by incubation for 24 h. Cells were lysed and subjected to ChIP using normal mouse IgG (IgG) or a mouse anti-p53 antibody (p53). Immunoprecipitated DNA was used as the template for real-time PCR quantification of the p53-binding sites on the p21^WAF1^ (C) or PUMA (D) promoters. Values are mean ± SEM for triplicates.

It has been demonstrated that acetylation of p53 by Tip60 stimulates binding of p53 to the PUMA promoter [18]. To examine whether the p38α-Tip60 pathway contributes to the ability of p53 to bind to the PUMA promoter, we tested the effect of p38α and Tip60 knockdown on the p53 occupancy on the PUMA promoter following DNA damage by ChIP assay. Chromatin DNA associated with p53 was immunoprecipitated from control cells or cells transduced with p38α or Tip60 shRNA after γ-radiation, and quantified by real-time PCR using primers amplifying the region from −1342 to −1449 of the PUMA promoter, which encompasses the p53 binding sites [3]. DNA damage increased binding of p53 to the PUMA promoter; however, the γ-radiation-induced p53 occupancy on the PUMA promoter was reduced in cells expressing either p38α shRNA (Figure 6B) or Tip60 shRNA (Figure 6D), as compared to the control cells expressing the GFP shRNA. These results indicate that the p38α-Tip60 pathway regulates the p53 activity in PUMA induction by stimulating p53 binding to the PUMA promoter in response to DNA damage.

We also analyzed the effect of p38α and Tip60 knockdown on p53 binding to the p21^WAF1^ promoter by the ChIP assay, using primers amplifying the p53 binding site-containing region (from -1463 to -1271) of the p21^WAF1^ promoter [30]. Whereas the Tip60 shRNA reduced DNA damage-induced binding of p53 to the PUMA promoter but not that to the p21^WAF1^ promoter (Figure 6C and 6D), the p38α shRNA abrogated p53 binding to both promoters in response to γ-radiation (Figure 6A and 6B). This again suggests that although Tip60 acetylation is only required for the ability to p53 to bind to and stimulate the transcription from the PUMA promoter, p38α activates another pathway in addition of Tip60-p53-PUMA, which mediates the induction of the p21^WAF1^ promoter by p53 upon DNA damage.

### Activated p38α directly phosphorylates Tip60-T158 *in vitro* and induces Tip60-T158 phosphorylation, p53-K120 acetylation, PUMA expression and apoptosis in cells

**Figure 7:**
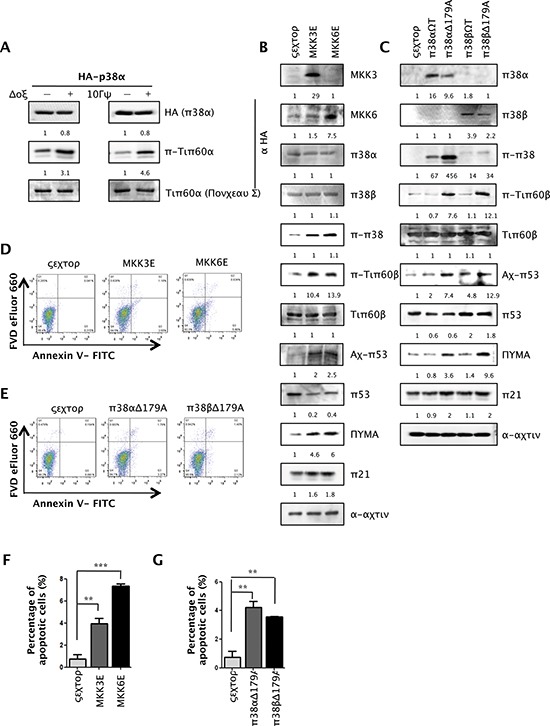
Activated p38α directly phosphorylates Tip60-T158 *in vitro* and induces Tip60-T158 phosphorylation, p53-K120 acetylation, PUMA expression and apoptosis in cells **(A)** Immunoprecipitation-coupled Kinase Assays for p38α. HA-p38α was immunoprecipitated from U2OS cells transduced with HA-p38α and treated with 1 μM of Dox for 36 h (left panels) or 10 Gy of γ-radiation followed by incubation for 48 h (right panels), and then incubated with recombinant Tip60α in the presence of cold ATP. Immunoprecipitated HA-p38α and Tip60-T158 phosphorylation were detected by Western blot using an anti-HA antibody and an anti-Tip60pT158 antibody, respectively. Input of recombinant Tip60α was stained by Ponceau S. **(B)** Western blot analysis of U2OS cells transduced with MKK3E, MKK6E or vector (Babe-puro), detecting MKK3, MKK6, p38α, p38β, p-p38, p-Tip60-T158, Tip60, ac-p53-K120, p53, PUMA, p21^WAF1^ and actin. Cells were lysed on day 3 post MKK3/6E transduction after selection of transduced cells. **(C)** Western blot analysis of U2OS cells transduced with wild-type (p38αWT, p38βWT) or indicated active mutant of p38 isoforms (p38αD179A, p38βD179A) or vector (Babe-puro), detecting p38α, p38β, p-p38, p-Tip60-T158, Tip60, ac-p53-K120, p53, PUMA, p21 and actin. Cells were lysed on day 3 post p38 transduction after selection of transduced cells. **(D)** FACS analysis of U2OS cells transduced with MKK3E, MKK6E or vector. Cells were collected on day 3 post MKK3/6E transduction after selection of transduced cells, and stained with a FITC-conjugated anti-Annexin-V antibody and FVD eFlour 660. **(E)** FACS analysis of U2OS cells transduced with wild-type (p38αWT, p38βWT) or indicated active mutant of p38 isoforms (p38αD179A, p38βD179A) or vector. Cells were collected on day 3 post p38 transduction after selection of transduced cells, and stained with a FITC-conjugated anti-Annexin-V antibody and FVD eFlour 660. **(F)** Quantification and statistical analysis of the data in D. The percentage of apoptotic cells was quantified as the percentage of FITC-positive cells in the gated area. Values are mean ± SEM for triplicates. **(G)** Quantification and statistical analysis of the data in E. The percentage of apoptotic cells was quantified as the percentage of FITC-positive cells in the gated area. Values are mean ± SEM for triplicates.

To directly assess Tip60 phosphorylation by p38α during DNA damage, we performed *in vitro* kinase assays with immunoprecipitated p38α using recombinant Tip60 as substrate. After U2OS cells transduced with HA-p38α were treated with Dox or γ-radiation, HA-p38α was immunoprecipitated and incubated with recombinant Tip60. Tip60 phosphorylation was assessed by Western blotting using the antibody against Tip60pT158. We found that phosphorylation of Tip60-T158 was increased by p38α isolated from Dox- or γ-radiation-treated cells over that from untreated cells (Figure 7A), indicating that DNA damage induces the protein kinase activity of p38α towards Tip60-T158.

We further determined the consequence of constitutive activation of p38 on Tip60, p53, PUMA and apoptosis, using constitutively active mutants of the p38 upstream kinases MKK3 and MKK6 (MKK3E and MKK6E), and a constitutively active mutant of p38α (p38αD179A) [27, 31]. Ectopic expression of MKK3E, MKK6E or p38αD179A increased activating phosphorylation of p38, and at the same time, induced Tip60-T158 phosphorylation, p53-K120 acetylation, PUMA expression, whereas the wild type p38α had no effect (Figure 7B, 7C). MKK3E, MKK6E or p38αD179A enhanced apoptosis in U2OS cells as compared to the vector controls (Figure 7D, 7E and 7F, 7G).

Therefore, these results indicate that DNA damage induces the protein kinase activity of p38α towards Tip60, and that in cells, activated p38α mediates Tip60-T158 phosphorylation and subsequent acetylation of p53-K120 by Tip60, leading to p53-mediated apoptosis.

### p38β also mediates Tip60-T158 phosphorylation, p53-K120 acetylation, PUMA expression and apoptosis in response to DNA damage

**Figure 8:**
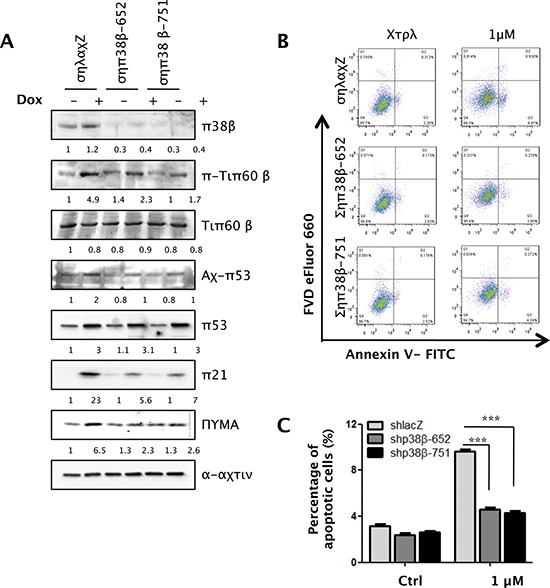
p38β is also essential for Tip60-T158 phosphorylation, p53-K120 acetylation, PUMA expression and apoptosis in response to DNA damage **(A)** U2OS cells transduced with shRNA for GFP (shGFP) or p38β (shp38β-652 or -751) were treated with 1 μM of Dox for 36 h. Cell lysis was subjected to Western blot analysis detecting the indicated proteins. **(B)** U2OS cells transduced with shRNA for GFP (shGFP) or p38β (shp38β-652 or -751) were treated with 1 μM for 36 h. Cells were collected, stained with an FITC-conjugated anti-Annexin-V antibody and FVD eFlour 660 and analyzed by FACS. **(C)** Quantification and statistical analysis of the data in B. The percentage of apoptotic cells was quantified as the percentage of FITC-positive cells in the gated area. Values are mean ± SEM for triplicates.

Interestingly, ectopic expression of a constitutively active mutant of p38β (p38βD179A) also induced Tip60-T158 phosphorylation, p53-K120 acetylation, PUMA expression and apoptosis, suggesting a possible involvement of p38β in this pathway (Figure 7C, 7E and 7G). We thus investigated whether p38β is also essential for the induction of the Tip60-p53-PUMA pathway and apoptosis by DNA damage. We examined the effect of p38β knockdown in U2OS cells during Dox-induced DNA damage. In contrast to the control lacZ shRNA, the p38β shRNAs (shp38β-652 and shp38β-751) knocked down p38β expression, greatly reduced the induction of Tip60-T158 phosphorylation and p53-K120 acetylation, and also decreased the induction of PUMA protein by Dox (Figure 8A).

We further analyzed the percentage of Annexin V-positive apoptotic cells by flow cytometry in Dox-treated cells with p38β knockdown. When incubated with 1 μM of Dox, 9% of control cells were apoptotic, whereas p38β knockdown reduced the percentage of apoptotic cells to 4% (Figure 8B and 8C). This finding confirms that p38β is required in DNA damage-induced apoptosis.

### p38α is essential for DNA damage-induced Tip60-T158 phosphorylation, p53-K120 acetylation, PUMA expression and apoptosis in primary human fibroblasts

**Figure 9:**
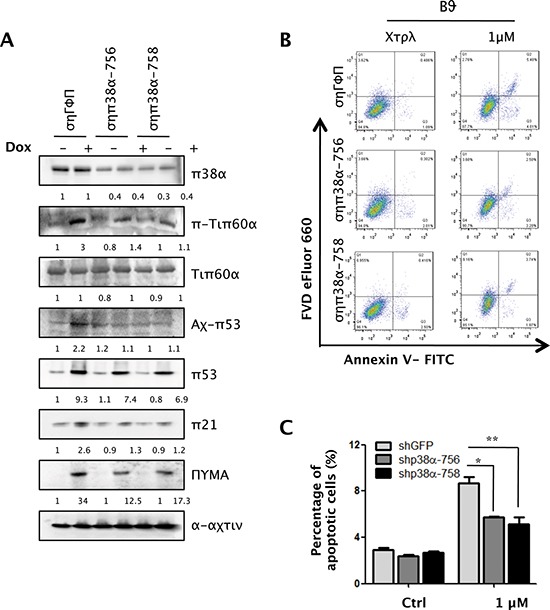
p38α is essential for DNA damage-induced Tip60-T158 phosphorylation, p53-K120 acetylation, PUMA expression and apoptosis in primary human fibroblasts **(A)** BJ cells transduced with shRNA for GFP (shGFP) or p38α (shp38α-756 or -758) were treated with 1 μM of Dox for 24 h. Cell lysis was subjected to Western blot analysis detecting the indicated proteins. **(B)** BJ cells transduced with shRNA for GFP (shGFP) or p38α (shp38α-756 or -758) were treated with 1 μM for 24 h. Cells were collected, stained with an FITC-conjugated anti-Annexin-V antibody and FVD eFlour 660 and analyzed by FACS. **(C)** Quantification and statistical analysis of the data in B. The percentage of apoptotic cells was quantified as the percentage of FITC-positive cells in the gated area. Values are mean ± SEM for triplicates.

To investigate whether the p38-Tip60-p53-PUMA signaling pathway is essential in DNA-damage induced apoptosis in the other cell lines, we knocked down p38α in primary BJ human fibroblast cells using shRNA. Consistent with the finding in U2OS cells, downregulation of p38α by shRNAs (shp38α-756 or shp38α-758) decreased the phosphorylation Tip60 at T158, acetylation of p53 at K120 and expression of PUMA and p21^WAF1^ induced by 1 μM of Dox (Figure 9A). Moreover, p38α knockdown reduced the percentage of apoptotic cells from nearly 8% to 5% in BJ cells treated with Dox (Figure 9B and 9C). Thus, the p38-Tip60-p53-PUMA pathway operates in both cancer cells and normal cells to mediate DNA damage-induced apoptosis.

### Phosphorylation of Tip60 at T158 by p38 is required for p53-K120 acetylation, PUMA induction and apoptosis after DNA damage

**Figure 10:**
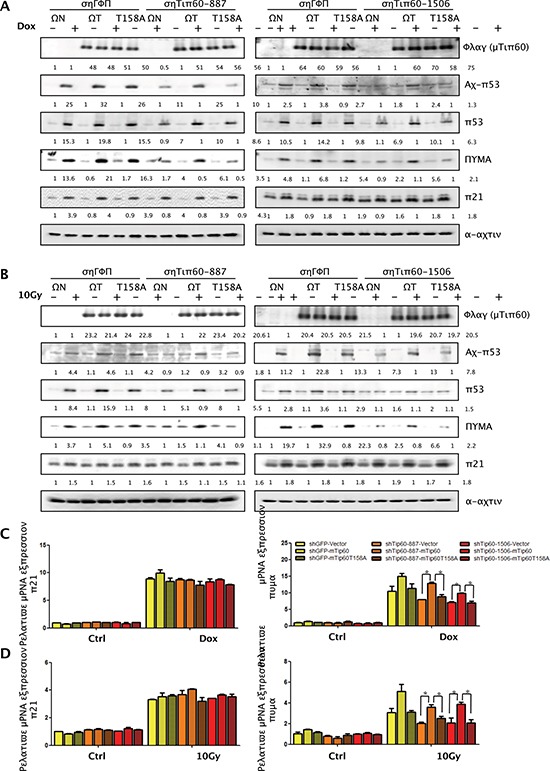
Phosphorylation of Tip60 at T158 by p38α is required for p53-K120 acetylation and PUMA induction after DNA damage **(A–B)** U2OS cells were co-transduced with shRNA for GFP (shGFP) or Tip60 (shTip60-887 or -1506) and vector (WN), wild type mouse Tip60 (WT) or mutant mouse Tip60 (T158A). Cells were treated with 1 μM of Dox for 36 h (A) or 10 Gy of γ-radiation followed by incubation for 24 h (B). Cell lysis was subjected to Western blot analysis detecting the indicated proteins. **(C–D)** U2OS cells were co-transduced with shRNA for GFP (shGFP) or Tip60 (shTip60-887 or -1506) and vector (WN), wild type mouse Tip60 (WT) or mutant mouse Tip60 (T158A). Cells were treated with 1 μM of Dox for 36 h (C) or 10 Gy of γ-radiation followed by incubation for 24 h (D) mRNA levels of PUMA (right panels) and p21^WAF1^ (left panels) were detected by real-time PCR. Values are mean ± SEM for triplicates.

We showed previously that p38-mediated Tip60 phosphorylation at T158 is required for the ability of Tip60 to mediate oncogene-induced senescence [20]. We thus investigated the functional relevance of this phosphorylation to DNA damage-induced responses, including p53 acetylation at K120, PUMA expression, and apoptosis. As shown previously, Tip60 shRNAs (shTip60-887 and -1506) reduced induction of p53 acetylation at K120 and PUMA expression by 1 μM or 3 μM of Dox or 10 Gy of γ-radiation (Figure 10A–10D). Ectopic expression of murine Tip60, which could not be knocked down by human Tip60 shRNA (Figure, 10A and 10B), restored DNA damage-induced p53-K120 acetylation (Figure 10A and 10B) and expression of PUMA at both protein level (Figure 10A and 10B) and mRNA level (Figure 10C and 10D) in U2OS cells expressing human Tip60 shRNA, indicating that murine Tip60 can functionally replace human Tip60 in these responses to DNA damage. In contrast, murine Tip60 carrying the T158A mutation, which cannot be phosphorylated by p38, failed to restore the induction of p53-K120 acetylation and PUMA expression by DNA damage in the presence of the human Tip60 shRNA (Figure 10A–10D). Therefore, phosphorylation of Tip60 at T158 is essential for the ability of Tip60 to mediate DNA damage-induced p53-K120 acetylation and PUMA expression. Neither Tip60 shRNA nor ectopic expression of murine Tip60 altered induction of p21^WAF1^ by DNA damage (Figure 10A and 10B), confirming that Tip60 does not play a critical role in the regulation of p21^WAF1^ expression in DNA damage.

**Figure 11:**
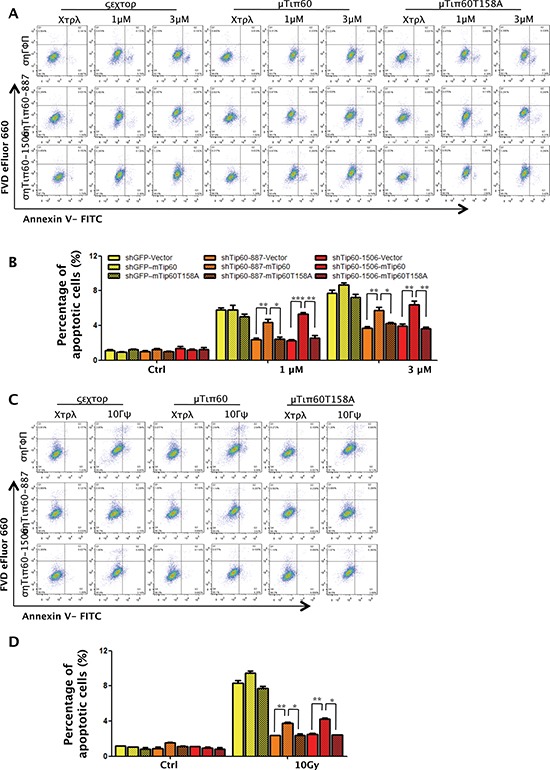
Phosphorylation of Tip60 at T158 by p38α is required for apoptosis induction in response to DNA damage **(A and C)** U2OS cells were co-transduced with shRNA for GFP (shGFP) or Tip60 (shTip60-887 or -1506) and vector (WN), wild type mouse Tip60 (WT) or mutant mouse Tip60 (T158A), and treated with 1 μM or 3 μM of Dox for 24 h (A) or 10 Gy of γ-radiation followed by incubation for 24 h (C). Cells were collected, stained with a FITC-conjugated anti-Annexin-V antibody and FVD eFlour 660, and analyzed by FACS. **(B and D)** Quantification and statistical analysis of the data in A (B) or C (D). The percentage of apoptotic cells was quantified as the percentage of FITC-positive cells in the gated area. Values are mean ± SEM for triplicates.

We further explored the importance of phosphorylation of Tip60 at T158 in DNA damage-induced apoptosis. While Tip60 shRNA decreased the percentage of apoptotic cells induced by 1 μM or 3 μM of Dox or 10 Gy of γ-radiation, ectopic expression of wild type murine Tip60 restored induction of apoptosis in U2OS cells expression human Tip60 shRNA after DNA damage; however, the T158A mutant of murine Tip60 failed to restore apoptosis abrogated by human Tip60 shRNA (Figure 11A–11D).

Taken together, these findings indicate that p38-mediated Tip60 phosphorylation at T158 plays an essential role in p53 acetylation at K120 and activation, induction of PUMA expression and induction of apoptosis during DNA damages.

## DISCUSSION

In a previous study, we identified p38 as a Tip60 kinase that induces the acetyltransferase activity of Tip60 by phosphorylating Tip60 at T158 [20]. In that same study, we demonstrated that upon phosphorylation and activation by p38, Tip60 mediates oncogene-induced senescence through acetylation of a downstream substrate PRAK. Tip60 is a multifunctional acetyltransferase that has been shown to mediate multiple cellular processes by acetylating different substrate proteins [20, 24, 32]. One important function of Tip60 is to mediate DNA damage-induced apoptosis by acetylating p53 at K120 and thus stimulating the ability of p53 to induce PUMA [14]. We thus investigated the effect of p38-mediated Tip60 phosphorylation on the p53-PUMA pathway during DNA damage-induced apoptosis in the current study. We showed that p38 activation, Tip60-T158 phosphorylation, and p53-K120 acetylation are induced with similar kinetics by DNA damage. p38α is essential for DNA damage-induced Tip60 phosphorylation at T158. In addition, both p38 and Tip60 are essential for p53 acetylation at K120, binding of p53 to the PUMA promoter, PUMA expression and apoptosis induced by DNA damage. Moreover, DNA damage induces the protein kinase activity of p38α towards Tip60-T158, and constitutive activation of p38 leads to increases in Tip60-T158 phosphorylation, p53-K120 acetylation, PUMA expression and apoptosis in cells. Furthermore, the Tip60-T158A mutant that cannot be phosphorylated by p38 failed to mediate p53-K120 acetylation, PUMA induction, and apoptosis following DNA damage. These results establish that Tip60-T158 phosphorylation by p38 plays an essential role in engaging the Tip60 activity required for inducing the p53-PUMA pathway that ultimately leads to apoptosis in response to DNA damage. These findings are consistent with previous reports showing that DNA damage induced apoptosis requires activation of p38 [33–34].

Both p38α and Tip60 have been shown to have tumor suppressing activities [21, 35–36]. Deletion of either p38α or Tip60 accelerates cancer development in mouse. Since p53-mediated apoptosis is an efficient cellular process that eliminates cells with potential oncogenic mutations resulted from unrepaired DNA damage, the essential role of p38α and Tip60 in DNA damage-induced apoptosis provides a mechanistic basis underlying the tumor suppressing function of these two proteins. Deletion of p38α and Tip60 may disrupt apoptosis of cells with unrepaired DNA, leading to accumulation of cells with oncogenic alterations and enhanced cancer development.

Consistent with the report that acetylation of p53-K120 by Tip60 only stimulates the ability of p53 to induce PUMA, but not p21^WAF1^, Tip60 shRNA reduced DNA damage-induced binding of p53 to the PUMA promoter and PUMA expression, but did not alter the binding of p53 to the p21^WAF1^ promoter or p21^WAF1^ expression. The T158A mutation of Tip60 also only abrogated the ability of Tip60 to mediate the induction of PUMA after DNA damage, without affecting p21^WAF1^ induction, suggesting that phosphorylation of Tip60 by p38 specifically regulates the p53-PUMA axis and apoptosis. In contrast, p38α knockdown abrogated binding of p53 to both PUMA and p21^WAF1^ promoters and reduced induction of both PUMA and p21^WAF1^ upon DNA damage. This indicates that in addition to phosphorylating Tip60 and promoting its activity towards the p53-PUMA axis, p38 activates another Tip60-independent pathway that stimulates the ability of p53 to induce p21^WAF1^. Indeed, it has been shown that p38 can regulate p53 activity by directly phosphorylating p53 at S33 and S46 in response to UV radiation [37]. In addition, p38 can phosphorylate a mRNA binding protein HuR at Thr118 in response to DNA damage, leading to accumulation of HuR in cytoplasma, which binds to and stabilizes the mRNA of p21^WAF1^ [38]. It is thus likely that p38 regulates DNA damage-induced apoptosis and cell cycle arrest through multiple pathways.

In addition to direct acetylation of p53 at K120, Tip60 has also been shown to regulate the activity of p53 as a transcriptional coactivator on p53 target promoters. While our data clearly indicate that phosphorylation of Tip60 by p38 contributes to p53 activation through direct acetylation of K120, further studies are needed to investigate the role of p38-mediated Tip60 phosphorylation in the function of Tip60 as a transcriptional coactivator of p53. Moreover, another member of the MYST family of acetyltransferases, hMOF, also phosphorylates p53 at K120. It will be interesting to investigate whether hMOF is phosphorylated and activated by p38 and contributes to p53 activation following DNA damage.

Although our studies clearly identify p38α as the p38 isoform that phosphorylates Tip60-T158 and mediates the subsequent acetylation of p53-K120 and p53-mediated apoptosis following DNA damage, our data indicate that p38β is also involved in this pathway. Ectopic expression of the constitutively active mutants of both p38α and p38β induced Tip60-T158 phosphorylation, p53-K120 acetylation, PUMA expression and apoptosis, whereas both p38α and p38β shRNAs abrogated these same changes induced by DNA damage. We propose that p38α and p38β have the same function in this pathway and mediate DNA damage-induced apoptosis through an identical mechanism. The knockdown of either p38α or p38β alone reduces the amount of total p38 in cells, thus leading to a decrease in the signaling strength of the p38-Tip60-p53-PUMA pathway and abrogation of apoptosis induction. Further experiments are needed to define the role of the other p38 isoforms in Tip60 phosphorylation and p53-mediated apoptosis in response to DNA damage. Indeed, a previous report showed that all 4 p38 isoforms are activated by γ-radiation in U2OS cells [39]. Using dominant negative mutants of p38 isoforms, this paper further demonstrated that p38γ, but not the other isoforms, is required for γ-radiation-induced G2 cell cycle arrest. It is thus possible that various p38 isoforms contribute to different aspects of the DNA damage response, such as apoptosis and cell cycle arrest.

## MATERIALS AND METHODS

### Cell culture

U2OS cells and LinX-A retroviral packaging cells were maintained in Dulbecco's modified eagle's medium supplemented with 10% fetal calf serum, sodium pyruvate, and antibiotics. 293T cells were grown in DMEM with 10% fetal calf serum, sodium pyruvate, glutamine, and antibiotics. BJ primary human fibroblasts were maintained in Minimum Essential Medium supplemented with 10% fetal calf serum, nonessential amino acids, glutamine, and antibiotics.

### Plasmids

Oligonucleotides for small hairpin RNAs (shRNAs) targeting p38β-652 (AAAAGCATTACAACCAA ACAGTGTTGGATC CAACACTGTTTGGTTGTAAT), p38β-751 (AAAAGCTGAAGCG CATCATGGAATT GGATCCAAT TCCATGATGCGCTTCA), and LacZ (AAAA-GCAGTTATCTGGAAGATCAGG-TTGGATCCAA-CCTGATCTTCCAGA TAACTGC) were cloned into pLV-EF1α-Puro lentiviral expression vectors (Biosettia, San Diego, CA) according to the manufacturer's protocol. Retroviral vectors encoding shRNAs targeting p38α [27], Tip60 [20] and p53 [40], expression vectors for mTip60 and mTip60T158A [20], and those for HA-p38α, MKK3E, MKK6E [40], as well as wild type and constitutive active mutants of p38 isoforms [27] were described previously.

### Retrovirus- and lentivirus-based gene transduction

Recombinant retroviruses were packaged and transduced into U2OS or BJ cells as previously described [41]. Recombinant pLV-EF1α-Puro lentiviruses were packaged in 293T cells following the manufacturer's protocol (Biosettia, San Diego, CA) and transduced into U2OS cells as previously described [42]. Transduced cells were purified with 80 μg/ml hygromycin B, and/or 2μg/ml puromycin.

### Analysis of apoptosis

U2OS or BJ cells were seeded into 15cm plates at a density of 2 × 10^6^ cells/plate. After treated with 1 μM or 3 μM Doxorubicin (Cat# 65-0864-14, Sigma, MO) for 24 h or exposure to 10 Gy γ-radiation followed by incubation for 16 h or 72 h, cells were harvested by trypsinization (Cat# 17-160E, Lonza, NJ), and combined with cells collected from suspension, after which cells were collected by centrifugation, washed with PBS, stained with Fixable Viability Dye eFluor® 660 (Cat# 65-0864-14, eBioscience, CA) for 30 min, and then wash with PBS and Annexin-V binding buffer (Cat# 88-8005, eBioscience, CA). 5μl of FITC-conjugated Annexin-V (Cat# 88-8005, eBioscience, CA) were added and incubated with cells at room temperature for 15 min. The percentage of FITC-positive apoptotic cells were determined by flow cytometry. Each experimental point was performed in triplicates.

### Western blot analysis

U2OS or BJ cells were seeded into 15cm plates at a density of 2 × 10^6^ cells/plate and treated with indicated dosages of Doxorubicin or γ radiation. Cell lysates were prepared in RIPA buffer described previously [43]. Cleared cell lysates were subjected to SDS-PAGE using 10 or 15% polyacrylamide gel and transferred to nitrocellulose membranes. The primary antibodies were from Cell Signaling (p38-pT180Y182, p38α and HA-tag), Santa Cruz (p53, p21^WAF1^), Prosci (PUMA), Abcam (ac-p53-K120) or Sigma (α-actin). The rabbit anti-Tip60 and p-Tip60-T158 antibody were raised in our lab [20].

### Quantitative real-time PCR

RNA was isolated from cells by using TRIzol reagent and converted to cDNA using iScript Reverse Transcription Supermix (Cat# 170-8840, Bio-Rad, CA) according to the manufacturer's protocol. Quantitative real-time reverse transcription-PCR (qRT-PCR) was performed using Sso Advanced SYBR green Supermix (Cat# 172-5271, Bio-Rad, CA) on a CFX96 real-time system (Bio-Rad). Signals were normalized to that of a housekeeping gene, β-actin. The primers used were 5′-TCAACGCACAGTACGAGCG-3′ and 5′-TGGGTAAGGGCAGGAGTCC-3′ for PUMA, 5′-TGTCACTGTCTTGTACCCTTG-3′ and 5′-GGCGTTTGGAGTGGTAGAA-3′ for p21, 5′-GGCATCCACGAAACTACCTT-3′ and 5′-CTCGTCATACTCCTGCTTGC-3′51  for β-actin.

### ChIP assays

U2OS cells were seeded into 15cm plates at a density of 2×10^6^ cells/plate and treated with 10Gy of γ radiation for 16 h or 72 h. ChIP assays were performed as described before [41]. After sonication, 10% of each sample was saved as total input, 45% of each sample was incubated with 5 μg of anti-p53 antibody (Cat# sc-126, Santa Cruz, TX), and the remaining 45% of each sample was incubated with 5 μg of normal mouse IgG (Cat# sc-2025, Santa Cruz, TX) at 4°C overnight and then with 50 μl (bead volume) of Pure Proteome proteinG magnetic beads (Cat# LSKMAGG02, Millipore, CA) at 4°C for 4 h. After wash and reverse crosslinking, DNA was extracted with phenol-chloroform, precipitated with ethanol, and dissolved in H_2_O. 2μl of ChIP material and 1μl of 1:10 dilution of total input were quantified by real-time PCR using primers amplifying the region from-1449 to -1342 of the PUMA promoter [3–4] (5′-TCCTCCTTGCCTGGGCTAG-3′ and 5′-GCGGACAAGTCAGGACTTGC-3′) or those amplifying the region from -1463 to -1271 of the p21^WAF1^ promoter (5′-GGGTCTGCTACTGTGTCCTC -3′and 5′-TTGGTGCAGCTACAATTACTG-3′). All the ChIP-qPCR data were normalized to the Input-qPCR data following Percent Input Method [44].

### Recombinant proteins

Recombinant His-Tip60α was prepared as described previously [20].

### Immunoprecipitation-coupled kinase assays for p38α

U2OS cells were lysed in a buffer containing 50 mM HEPES, pH 7.5, 2.5 mM EGTA, 1 mM EDTA, 1% Triton X-100, 150 mM NaCl, 10% glycerol, 1 mM phenylmethylsulfonyl fluoride, 50 mM NaF, 1 mM sodium vanadate, 1 mM β-glycerophosphate, 1 mM dithiothreitol, and Complete protease inhibitors. 500μg of lysate were incubated with 2.5 μg of p38*α* rabbit polyclonal antibody or HA-11 mouse monoclonal antibody (Covance) at 4°C overnight, after which 30 μl of Protein A/G agarose or Protein G agarose (Pierce) were added and incubated for additional 2 h. The beads were washed two times with 1 ml of lysis buffer and two times with kinase buffer (50 mM HEPES, pH 7.5, 0.5 mM EGTA, 10 mM MgCl2, 0.1 mM phenylmethylsulfonyl fluoride, 1 mM NaF, 0.1 mM sodium vanadate, 0.1 mM β-glycerophosphate, and 1 mM dithiothreitol). The reactions were performed in 20 μl of kinase buffer (above) with 20 μM cold ATP, and 10 μg of recombinant His-Tip60α at 30°C for 45 min. The reactions were stopped by 7 μl of 4 × Laemmli buffer, heated at 95°C, and the supernatants were subjected to Western blot analysis. HA-p38 was detected by a rabbit HA antibody (Cell signaling) and p38α was detected by a mouse p38α antibody (Cell signaling).

### Statistical analysis

Values were expressed as Means ± S.E.M. Significance were determined by Student's *t*-test. A value of *p* < 0.05 was used as the criterion for statistical significance. * indicates significant difference with *p* < 0.05, ** indicates significant difference with *p* < 0.01, *** indicates significant difference with *p* < 0.001.
